# Paravertebral Mass in a Patient with Hemolytic Anemia: Computed Tomographic Findings

**DOI:** 10.1155/2010/724279

**Published:** 2010-03-25

**Authors:** Juliana França Carvalho, Edson Marchiori, Gláucia Zanetti, Claudia Mauro Mano, Branca Sarcinelli-Luz, Flávia Gavinho Vianna, Carla Assed, Isabella Guedes Santos, Alair Augusto S. M. D. Santos, Alberto Domingues Vianna

**Affiliations:** Department of Radiology, Faculty of Medicine, Fluminense Federal University, CEP 24030.215. Niterói, Rio de Janeiro, Brazil

## Abstract

Extramedullary hematopoiesis is characterized by the presence of hematopoietic tissue outside of the bone marrow and is typically associated with chronic hemolytic anemias. Intrathoracic extramedullary hematopoiesis is a rare and usually asymptomatic condition. The authors report a case of a 57-year-old man with intrathoracic extramedullary hematopoiesis and hereditary spherocytosis. Clinical and laboratory evaluation, together with radiological findings, are described. The diagnosis of the disease was confirmed by tissue biopsy.

## 1. Introduction

Extramedullary hematopoiesis (EMH) is a physiologic compensatory mechanism for bone marrow dysfunction. It usually develops in congenital hemolytic anemias or in acquired marrow replacement disorders [1, 2]. Although most frequently found in the liver, spleen, and lymph nodes, any organ can be involved [2–4]. Intrathoracic extramedullary hematopoiesis (IEH) is a rare entity and it commonly manifests as multiple lobulated soft-tissue masses in the paravertebral regions [1, 3]. Thoracic involvement is usually asymptomatic [1, 3]. Noninvasive studies, such as conventional radiology, computed tomography (CT), magnetic resonance imaging (MRI), or scintigraphy, can establish the diagnosis when characteristic radiological findings are present in the proper clinical setting [3, 5]. Because of the inherent risk of bleeding complications, tissue biopsy should be reserved for cases in which other causes of posterior mediastinal masses cannot be excluded [1, 4, 6]. 

We describe a case of IEH in a patient with previously unknown hereditary spherocytosis, simulating posterior mediastinal tumor. The final diagnosis was confirmed by tissue biopsy, and a splenectomy was then performed.

## 2. Case Presentation

A 57-year-old Brazilian Afro-American man presented to a hospital with a two-year history of weakness and paleness, associated with occasional chest pain. The patient also reported a weight loss of 5 Kg in that period and pain in the right upper abdomen. He had no significant past medical history. At that time, the diagnosis of anemia was made and he was transfused with packed red blood cells. The patient was then referred to our hospital for further investigation. 

Physical examination at the time of the admission revealed a pale, anicteric man, with a hyperdynamic precordium and a systolic heart murmur on auscultation. The liver edge was palpable 12 cm below the right costal margin, with its left lobe 7 cm below the xiphoid process. There was also a marked painless splenomegaly. Clinical examination was otherwise unremarkable. 

Hematological investigation showed a red blood cell count of 2,900,000/mm^3^, a hematocrit value of 23%, and a hemoglobin level of 7.9 g/dL. Platelet count was 180,000/mm^3^ and reticulocyte count was 274,550/mm^3^ (9.5%). Peripheral smear revealed severe poikilocytosis and anisocytosis, with a significant number of spherocytes. Rare ovalocytes, schistocytes, and macrocytes were also detected, as well as giant platelets and some degree of policromasia. Serum biochemical analysis was as follows: aspartate aminotransferase 120 U/dL; alanine aminotransferase 100 U/dL; alkaline phosphatase 147 U/dL; gamma-glutamyl transpeptidase 93 U/dL; total proteins 6.40 g/dL; albumin 3.91 g/dL; globulin 2.49 g/dL; total bilirrubin 2.71 mg/dL; indirect bilirubin 2.08 mg/dL, and direct bilirubin level was 0.63 mg/dL. No erythrocyte autoantibodies were detected by the direct Coombs test. On osmotic fragility test, initial hemolysis occurred at NaCl concentrations of 0.54% (control at 0.48%) and final hemolylis at 0.30% (control at 0.30%). Based on the aforementioned data, the diagnosis of hereditary spherocytosis was made. 

**Figure 1:**
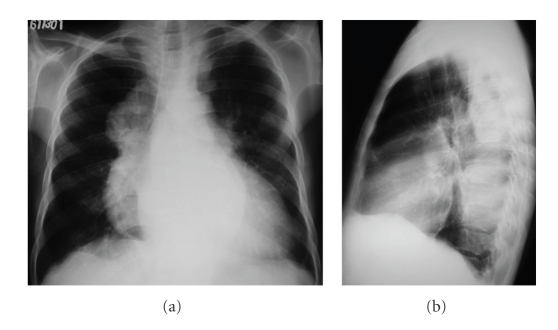
Anteroposterior (a) and lateral (b) chest radiographs showing large lobulated bilateral masses located in the retrocardiac region.

**Figure 2:**
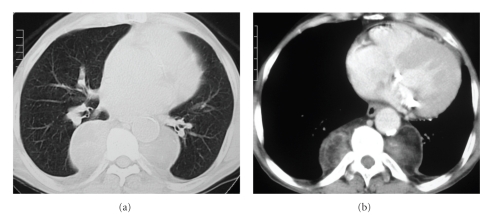
Computed tomography with pulmonary (a) and mediastinal (b) window settings demonstrates smooth heterogeneous (soft tissue and fat densities) bilateral masses in the paravertebral regions.

A chest radiograph (Figure 1) showed well-circumscribed, lobulated, soft-tissue masses located at the posterior mediastinal regions. For further evaluation, a chest CT scan (Figure 2) was performed and confirmed the presence of round, bilateral, heterogeneous, paravertebral masses as well as hepatosplenomegaly. Abdominal ultrasonography showed an enlarged homogeneous liver with regular edges and no focal lesions. The spleen was homogeneous and measured 23 cm. 

Thoracotomy was performed and tissue biopsy confirmed the final diagnosis of EMH. The patient underwent splenectomy one month later. Postoperative recovery was uneventful, and he was recommended for periodic controls.

## 3. Discussion

Extramedullary hematopoiesis occurs as a compensatory response in various congenital hemolytic anemias, such as thalassemia, sickle cell anemia and hereditary spherocytosis, as well as in myeloproliferative disorders, such as leukemia, lymphoma, myelodysplasia, and myelofibrosis [3, 5, 7, 8]. 

Although the most common sites of EMH are the liver, spleen, and lymph nodes, diffuse microscopic infiltration of kidney, adrenal gland, lung, pleura, skin, breasts, dura-mater, ovary, thymus, gastrointestinal tract, and central nervous system have also been reported in [2, 7, 8]. 

IEH is a rare and usually asymptomatic condition, although occasional symptoms may be associated with complications, as hemothorax, pleural effusion, or spinal cord compression [1, 6, 7]. 

The pathogenesis of intrathoracic EMH includes the extrusion of proliferating bone-marrow steam-cells through the thin cortex of the vertebral bodies and ribs into the subperiosteal region, promoted by the negative pressure of the thoracic cavity. Proliferation of embolized hematopoietic tissues from other areas, such as the spleen, can explain other heterotopic marrow foci [1, 2, 5, 8]. 

On conventional radiology, IEH presents as lobulated, smoothly marginated, paravertebral masses, usually multiple and bilateral and caudal to the sixth thoracic vertebra, without calcifications or bone erosion [2, 5, 8]. The absence of bone destruction is important for the differential diagnosis with other posterior mediastinal lesions, especially neurogenic tumors [5, 8]. Less frequently masses can also be found in the anterior mediastinum or in the pleura [1, 5]. Interstitial pulmonary involvement has also been reported in [3, 5]. 

CT scans show homogeneous soft-tissue attenuation masses, with characteristics similar to those described on chest X-rays and which may or may not be enhanced after the administration of intravenous contrast [1, 3, 5, 7–9]. This method demonstrates with accuracy the internal structure of the masses [5, 8]. Moreover, CT scanning is important for the detection of bone alterations related to the underlying disease, such as coarsening of the trabecular pattern and widening of the medullary cavities of the ribs [2, 3, 5]. 

MRI is the best method for the evaluation of spinal cord compression [6]. Technetium^99^ sulfur colloid radionuclide bone marrow scan may demonstrate activity in the mass [1, 9, 10]. 

When a patient with a previously diagnosed hematological disease presents the typical radiographic manifestations, the diagnosis of IEH can be safely established and there is no need of further invasive studies. Because of the highly vascular nature of the masses, biopsy and surgical resection should be avoided in order to prevent hemorrhage [4–7]. 

Treatment is not required unless complications are present. Hematopoietic tissue is radiosensitive and a low dose radiation therapy is effective in cases of medullary compression [1, 5, 7, 9]. Since the EMH foci represent the source of compensatory erythrocyte formation, surgical resection of the masses should only be performed in severe cases of spinal cord compression, which could lead to neurological sequel [1, 5, 8]. Symptomatic pleural effusion or hemothorax that persists after drainage can also be controlled with radiotherapy [5]. Splenectomy is well recommended for patients with hereditary spherocytosis [1, 8]. 

In conclusion, based on characteristic radiological and clinical findings, it is important to recognize the possibility of IEH in the differential diagnosis of mediastinal masses.

## List of Abbreviations

EMH: Extramedullary hematopoiesis
IEH: Intrathoracic extramedullary hematopoiesis
CT: Computed tomography
MRI: Magnetic resonance imaging.
